# Drivers of irrational use of antibiotics among children: a mixed-method study among prescribers and dispensers in Tanzania

**DOI:** 10.1186/s12913-022-08359-7

**Published:** 2022-07-29

**Authors:** Lilian Nkinda, Manase Kilonzi, Fatuma F. Felix, Ritah Mutagonda, David T. Myemba, Dorkasi L. Mwakawanga, Upendo Kibwana, Belinda J. Njiro, Harrieth P. Ndumwa, Rogers Mwakalukwa, Gerald Makuka, Samson W. Kubigwa, Alphonce I. Marealle, Wigilya P. Mikomangwa, Godfrey Sambayi, Peter P. Kunambi, Betty A. Maganda, Nathanael Sirili, Rashid Mfaume, Arapha Bashir Nshau, George M. Bwire, Robert Scherpbier, Elevanie Nyankesha, Pacifique Ndayishimiye

**Affiliations:** 1 School of Medicine, Muhimbili University of Health and Allied Sciences, P.O. Box 65001, Dar es Salaam, Tanzania; 2 School of Pharmacy, Muhimbili University of Health and Allied Sciences, P.O. Box 65001, Dar es Salaam, Tanzania; 3 School of Nursing, Muhimbili University of Health and Allied Sciences, P.O. Box 65001, Dar es Salaam, Tanzania; 4 Liwale District Hospital, P.O Box 28, Lindi, Tanzania; 5 School of Public Health and Social Sciences, Muhimbili University of Health and Allied Sciences, P.O. Box 65001, Dar es Salaam, Tanzania; 6 Regional Administrative Secretary, Dar Es Salaam Region, P.O. Box 5429, Dar es Salaam, Tanzania; 7 Pharmacy Council, Ministry of Health, Community Development, Gender, Elderly and Children, P.O. Box 31818, Dar es Salaam, Tanzania; 8 United Nations Children’s Fund, Bâtiment BIT, Route des Morillons 4, CH-1211 Geneva 22, Switzerland; 9 United Nations Children’s Fund, NY HeadQuarter, 3 United Nations Plaza, New York, NY 10017 USA; 10 School of Medicine and Pharmacy, College of Medicine and Health Sciences, University of Rwanda, P.O. Box 4285, Kigali, Rwanda

**Keywords:** Irrational antibiotic use, Practice, Tanzania, Children, Prescribers, Dispenser

## Abstract

**Background:**

Misuse of antibiotics has been associated with poor knowledge, attitude and practice (KAP). Therefore, this study aimed to assess if KAP of prescribers and dispensers could drive irrational use of antibiotics among children in Tanzania.

**Methods:**

A convergent parallel mixed-methods study design that employed quantitative and qualitative approaches was conducted in 14 regional referral hospitals (RRHs). A total of 108 participants, prescribers [54] and dispensers [54] working with the pediatric population in the respective regions participated in a quantitative survey, by filling the standard questionnaire while 28 key informant interviews were conducted with in-charges of units from the pharmacy and pediatric departments. Two key informants (prescriber and dispenser) were selected from each RRH.

**Results:**

Overall, among prescribers and dispensers, there was adequate knowledge; 81.5% and 79.6%, *p* = 0.53, those with positive attitudes were 31.5% and 81.5%, *p* < 0.001 and poor practices were among 70.4% and 48% *p* = 0.0312 respectively. Among prescribers, 14.8% agreed and strongly agreed that prescribing antibiotics that a patient did not need does not contribute to resistance. Moreover 19% disagreed to prescribe antibiotics according to local guidelines. Among dispensers, a-quarter of the dispensers thought individual efforts to implement antibiotic stewardship would not make a difference, 17% agreed and strongly agreed that antibiotics can treat viral infection and 7% agreed and strongly agreed antibiotics can be stopped upon resolution of symptoms. From qualitative interviews, both participants displayed an adequate understanding of multi-contributors of antibiotic resistance (AR) including polypharmacy, community self-medication, among others. Regardless, both professions declared to prescribed and dispensed antibiotics according to the antibiotics available in stock at the facility. Furthermore, prescribers perceived laboratory investigation took a long time, hence wasting their time. On the other hand, Dispensers reported not to provide adequate instruction to the patients, after dispensing antibiotics.

**Conclusions:**

Both prescribers and dispensers had adequate knowledge, few prescribers had positive attitudes and the majority had poor practices. Few dispensers had poor attitude and practice. These findings highlight the need to provide adequate training on antimicrobial stewardship and enforce regulation that foster appropriate medical practice.

**Supplementary Information:**

The online version contains supplementary material available at 10.1186/s12913-022-08359-7.

## Background

Antibiotic resistance (AR) is rampant and is considered a global health threat of the twenty-first century [1]. Current estimates indicate without effective actions, the continued rise of AR could result to 10 million deaths and cost up to 1 trillion USD every year by 2050 [2, 3]. According to the World Health Organization (WHO) definition, AR is a failure of bacteria to respond effectively to antibiotics that were originally potent for the treatment of infections caused by it [4].

The irrational use of antibiotics is a major factor fueling the development of AR [5]. According to the WHO estimates, more than half of all medicines are prescribed, dispensed, or sold inappropriately [6]. Available evidence shows that irrational use of antibiotics is rampant in low and middle-income countries whereby prescription without laboratory evidence and non-prescription sale is commonly practiced [7]. In Tanzania, a recent report has shown that more than 60% of patients in primary health care received antibiotics [8], higher than WHO recommendation of 20–26.8% [9, 10]. Moreover, almost one-third of all clients buying antibiotics in drug dispensing outlets do not have a prescription [8]. These findings demonstrate the role of health care workers on the irrational use of antibiotics.

Evidence from research agrees that one way to address antibiotics resistance (AR) is to consider the vital role played by healthcare professionals, especially prescribers and dispensers [11], as they are trained on the rational use of antibiotics, and are expected to champion AR stewardship programs [12]. Moreover, in several studies, the misuse of antibiotics has been associated with their poor knowledge, attitude, and practice (KAP) [13, 14]. Reports from Tanzania on rational use of antibiotics with reference to children are scarce, most confined in the Northern part of Tanzania [15–17], and others are focused on parents/caregivers [18, 19]. These reports show, poor practices by healthcare workers, that translates to antibiotic overdosing ranging from 60–87% [17] treatment of viral infection with antibiotics 28–69% [17, 20] and polypharmacy 15–30% [17, 20], however they cannot be generalized to other regions of Tanzania. This is because the northern zone is a relatively wealthy part of the country, with higher levels of literacy, health facilities are easily accessible to the community and are comparably equipped with diagnostics [16].

Moreover, although parents are believed to be a determining factor for appropriate or inappropriate use of antibiotics among children, their practices and most of what they know or do not know concerning antibiotics is influenced by health care workers. This is evidenced by findings from parents (*n* = 2802) in one of the largest study in 14 referral regional hospitals in Tanzania, whereby only 10% and 16% of parents had good knowledge and positive attitudes, and the majority (82%) had poor practices on rational use of antibiotics for children [18]. Despite this, 86% and 77% reported dispensers and prescribers respectively to be their main source of antibiotic information. Furthermore, half of them reported they would seek another prescriber if the initial dose did not give them antibiotics [18]. This shows a clear role of health care workers to control and mitigate the rampant use of antibiotics among children in Tanzania.

Therefore, this study was conducted to assess (KAP) of prescribers and dispensers and its influence on antibiotic use among children to fill the gap of evidence on antibiotic use among children in Tanzania Mainland.

## Methods

### Study design, study period and study site

A hospital-based cross-sectional design using convergent parallel mixed-methods (quantitative and qualitative) was conducted in 14 RRHs in Tanzania Mainland between June and September 2020. Two RRHs from each zone were selected to represent seven administrative zones in Tanzania Mainland. Selection of hospitals were based on regional socio-economic activities [21] such as livestock keeping, farming and mining as they are likely to play roles in antibiotics use [22]. The RRHs were selected as the representative of the health facilities [23] since they have capacity to implement rational use of antibiotics and offer a wide range of clinical services such as; pediatrics, outpatient and inpatient care, emergency services, diagnostics (laboratory, radiology and imaging) and pharmaceutical service and supply among others.

### Study population and Sampling technique

Purposive sampling was used to select participants for both quantitative and qualitative studies. For the quantitative data, all prescribers and dispensers working in RRH pediatric and pharmacy units respectively with at least a diploma level education were assessed upon signing the informed consent form. Prescriber with diploma were 8(15%), and dispensers with diploma were 25(46%). For the purpose of triangulation of the study population, participants in charge of the units in pediatric and pharmacy departments who had at least two years of experience in the administrative role were recruited to participate in the qualitative study. In this case, two informants (dispenser and prescriber) were recruited from each RRH to participate in the key informant interview. To limit response bias, the informants who participated in the qualitative study were excluded from participating in the quantitative study.

#### Professional education and roles in the context of Tanzania

A prescriber with a diploma is one level below a medical doctor (Bachelor's degree), during the study period, prescription was restricted according to the facility level, in which capacity to do culture and sensitivity was one of the criteria. Therefore, a diploma holder working at a RRH could prescribe all levels of antibiotics as a medical doctor would. A diploma in pharmacy or a pharmaceutical technician is one level below pharmacists (bachelor's degree holder). They assist pharmacists in compounding and dispensing activities in a pharmacy. In cases where both a pharmacist and technician are present, the pharmacist will review prescriptions, communicate with physicians for queries, and counsel patients, whereas the technician will mainly perform packaging, labeling, and compounding tasks. However, due to a shortage of healthcare workers in our country and many other LMICs, technicians may assume some pharmacist's responsibilities in centers where there are few or no pharmacists. This may also involve querying and making recommendations to physicians, although they are most often required to consult senior colleagues first, such as the head of department, or any pharmacist that can possibly be reached within a reasonable time. Their contribution to antimicrobial stewardship is therefore significant, whether that be in the form of assisting pharmacists or taking more pronounced, empowered and leading roles where pharmacists are not available.

### Sample size

A total of 108 prescribers (54) and dispensers (54) were assessed for the quantitative study. For qualitative data, 28 in-depth interviews among the 28 Prescribers and Dispensers in charge of the pediatric unit and pharmacy unit respectively were done.

### Data collection procedures

#### Quantitative data collection

Two different 5-point Likert scale structured questionnaires for dispensers and prescribers were adopted from the previous study [13, 14] after conducting thorough literature review. The questionnaire consisted of five major areas: i) general information and consent ii) social demographics of participants (prescribers and dispensers); iii) knowledge of the prescribers and dispensers’ iv) attitude of the prescribers and dispensers and v) practice of the prescribers and dispensers. Questions on knowledge assessed to what extent a participant knows about antibiotics, rational uses of antibiotics in children, and how AR occurs. The attitude of participants towards rational uses of antibiotics in children and AR were also assessed. Moreover, how their practice influenced irrational use of antibiotics, efforts taken as an individual and as a department to fight against AR. Furthermore, sources of where the participants got information related to antibiotics uses and AR were questioned. The questionnaires were translated to Swahili pre-tested if they answered the objectives of the study by randomly selecting 10 prescribers and 10 dispensers in a pilot study conducted in Dar es Salaam, region. The interview was done in Swahili and translated back to English for analysis.

#### Qualitative data collection

A semi-structured interview guide was developed following a comprehensive literature review. The questions were focused on knowledge of antibiotics, AR, and antibiotic stewardship. Also, the questions evaluated the attitude of prescribers and dispensers towards antibiotic stewardship, activities to fight against AR and continuing education of antibiotic resistance as means of fighting against AR. Furthermore, effort done by prescribers/dispensers in reducing AR, and antibiotic stewardship activities were assessed. Lastly, their recommendation on what could be improved in order to reduce AR and ways to accelerate the implementation of antibiotic stewardship was questioned. A total of 28 key informant interviews were conducted with prescribers and dispensers who were in charge of pediatric units using a semi-structured interview guide to explore what drives the irrational use of antibiotics. All interviews were conducted in Swahili language a native local language for both researchers and participants. Interviews were audio-recorded to capture the information provided by the participants. During the interview process field notes on verbal and non-verbal aspects were taken to complement the audio recorded information. Each interview lasted between 25 and 45 min.

### Data analysis

#### Quantitative analysis

Open Data Kit (ODK Software, USA) was used in data collection. Collected data were exported to Microsoft Excel Sheet (Redmond, WA) then to statistical package for social sciences version 25 (SPSS Software, Chicago Inc., USA) for coding and data analysis. For KAP, the scores were 1 for correct and 0 for wrong or uncertain/neutral responses on individual questions, respectively. The scores for poor knowledge was 0–40%, moderate knowledge was 50–70% and good knowledge was 80–100%. The scores for negative attitude was 0–40%, uncertainty or sometimes was 50–70% and positive attitude was 80–100%. The percentage score for practice was either poor or good if the score was 0–70% or 80–100%, respectively. Categorical variables such as age, sex and education level were presented using frequencies and percentages. The Association of demographic variables, education level, experience and KAP between prescribers and dispensers was computed using Chi-square test. The *p*-value of less than 0.05 was the measure of statistical significance.

#### Qualitative analysis

The audio recorded interviews and FGDs were first transcribed verbatim. The transcripts and field notes were read and re-read by six researchers to be familiar with the data and understand the participant`s accounts before the coding process. Thematic analysis was conducted following procedures delineated by Braun and Clarke [24]. Team-based coding was implemented for codebook development and data analysis [25, 26]. A codebook contaeining an initial list of codes for data analysis, based on our study objectives. We then refined the codebook from the themes, which emerged inductively during the analysis. Six researchers were involved in the coding process which was done in pairs to ensure reliability. The whole process of analysis was iterative. The discussion on the discrepancies was done with the entire team to reach agreements. New emergent codes were assigned as separate codes or as an expansion of the codes available in the initial codebook. The identified list of codes were assessed for commonalities and differences and grouped into sub-themes and themes. The final themes were then refined and discussed with the entire team and have been presented with respective participants' quotes.

## Results

### Quantitative findings

#### Participants’ socio-demographic

Most of the prescribers 75.9% (*n* = 41) had age between 26–35 years, similarly to dispensers 51.9% (*n* = 27), *p* = 0.0692. Most of the prescribers were graduates/ medical doctors 75.9% (*n* = 41) while for dispensers the majority were diploma holders/ pharmaceutical technicians 46.3% (*n* = 25), *p* < 0.001. Most of the participants in both groups 68.5% (*n* = 37) each, *p* = 0.01074 had the experience of 1 to 5 years of working with the pediatric population (Table 1).

**Table 1:** Socio-demographic characteristics of the prescribers and dispensers

		**Prescribers**	**Dispensers**	***P*-value**
**Variable**	Categories	n (%)	n (%)	
Age (years)	≤ 25	4 (7.4)	10 (19.2)	0.0692
	26 – 35	41 (75.9%)	27 (51.9)	
	36 – 45	5 (9.3%)	7 (13.5)	
	> 45	4 (7.4%)	8 (15.4)	
Sex	Male	33 (61.1)	35 (64.8)	0.8421
	Female	21 (38.9)	19 (35.2)	
Marital status	Married	25 (46.3)	30 (55.6)	0.3358
	Not married	29 (53.7)	24 (44.4)	
Professional education level	Postgraduate	5 (9.3)	0 (0.0)	< 0.001
	Graduate	41 (75.9)	16 (29.6)	
	Diploma	^a^8 (14.8)	^b^25 (46.3)	
	Certificate	0 (0.0)	13 (24.1)	
Experience working with pediatric patients (years)	< 1	9 (16.7)	1 (1.9)	0.01074
	1 – 5	37 (68.5)	37 (68.5)	
	> 5	8 (14.8)	16 (29.6)	

^a^Diploma in Clinical Medicine

^b^Diploma in Pharmacy (Pharmaceutical technician)

#### KAP on rational use of antibiotics among prescribers and dispensers

Overall KAP in relation to ASPs was assessed among 54 prescribers and 54 dispensers. Majority of the prescribers 81.5% (*n* = 44) and dispensers 79.9% (*n* = 43), *p* = 0.5278 had a good knowledge about antibiotics use in children. Regarding the attitude, 31.5% (*n* = 31.5) of the prescribers and 81.5% (*n* = 44) of the dispensers, *p* < 0.001 had positive attitudes. Poor practice was observed in the majority of the prescribers 70.4% (*n* = 38) while 51.9% (*n* = 28) of dispensers, *p* = 0.0312 had good practice (Table 2).

**Table 2:** Prescribers’ and dispensers’ KAP on rational use of antibiotics

		**Profession**		
**Variable**	**Categories**	**Prescriber, n (%)**	**Dispenser, n (%)**	***P-*values**
Knowledge	Poor	4 (7.4)	2 (3.7)	0.5278
	Moderate	6 (11.1)	9 (16.7)	
	Good	44 (81.5)	43 (79.6)	
Attitude	Negative	13 (24.1)	5 (9.3)	** < 0.001**
	Uncertain	24 (44.4)	5 (9.3)	
	Positive	17 (31.5)	44 (81.5)	
Practices	Poor	38 (70.4)	26 (48.1)	**0.0312**
	Good	16 (29.6)	28 (51.9)	

##### Attributes of poor knowledge among prescribers and dispensers

Poor to moderate knowledge among prescribers were attributed by 24% and 4% who agreed and strongly agreed that it was hard for them to choose the right antimicrobial. About 19% were unsure if resistance is a world-wide problem and 7.4% were not sure if antibiotics can be used for viral infection and 7.4% were uncertain if they had to know the resistance pattern of an antibiotic before prescribing it.

Poor to moderate knowledge among dispensers was attributed by 7.4% and 5.6% who agreed and strongly agreed that you can stop taking medication if symptoms resolved and 11% and 5.6% who agreed and strongly agreed that antibiotics can be used to treat viral infection (Additional files 1 and 2).

##### Attributes of negative attitudes among prescribers and dispensers

Negative attitudes among prescribers were contributed by 7.4% and 7.4% who agreed and strongly agreed that prescribing antibiotics while a patient does not need them does not contribute to resistance.

Negative attitudes among dispensers were attributed by 33%, who agreed that individual efforts on antibiotic stewardship have minimal impact on resistance (Additional files 1 and 2).

##### Attributes of poor practices among prescribers and dispensers

Poor practices among prescribers were attributed by 19% who disagreed to prescribe antibiotics according to local guidelines, 19% and 17% who agreed and strongly agreed to prescribe antibiotics according to the availability of the antibiotic at the facility.

Poor practices among dispensers were attributed by 29% who sometimes dispensed antibiotics without prescription and 7% and 6% who often and almost always dispensed without antibiotics respectively. About 11% never involved themselves in antibiotic awareness campaigns and 7.4% seldom /rarely educate patients on the use of antibiotics and resistance issues (Additional files 1 and 2).

#### Prescribers and dispensers’ source(s) of information about antibiotics

**Fig. 1:**
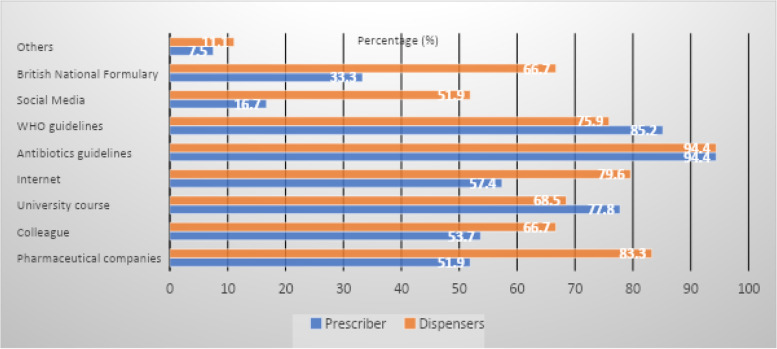
Prescribers and dispensers’ source(s) of information about antibiotics

The majority of prescribers and dispensers (94.4%) (Fig. 1) used antibiotics guidelines as their main source of information. Pharmaceutical companies were the second leading source of information among dispensers (83.3%) while WHO guidelines were the second prescribers’ preference (85.2%).

### Qualitative findings

#### Characteristics of key informants

In total 28 key informants (14 prescribers and 14 dispensers) were interviewed from 14 RRHs in Tanzania Mainland. Participants’ characteristics are summarized in (Table 3).

**Table 3:** Characteristics of key informants

Variable	Prescribers n(%)	Dispenser n(%)
**Age (years)**		
< 35	8(57)	10(71)
35 and above	6(43)	4(29)
**Sex**		
Male	5(36)	10(71)
Female	9(64)	4(29)
**Professional education level**		
Postgraduate	5(36)	0(0)
Graduate	9(64)	10(71)
Diploma	0(0)	4(29)
**Experience of working with pediatric patients (years)**		
2	4(29)	5(36)
3 and above	10(71)	9(64)

Prescriber means entitled to write antibiotics prescription, Dispenser means entitled to dispense antibiotics

#### Knowledge on antibiotics use in children and AR

##### Adequate knowledge of antibiotics use in children and AR

Prescribers and dispensers stated that they have adequate knowledge on the basics of antibiotics use in pediatric patients and the cause of AR. Their knowledge included but was not limited to correct knowledge on the group of antibiotics to be used in pediatric patients. They added that the adequate knowledge influenced how they interacted with the patients and thus influenced the rational use of antibiotics among the patients.

“…Patients should receive the prescribed right dosage, at the right time and at the recommended time interval …… for example; appropriate dose is when a patient takes one tablet three times a day and dosage is when a patient takes some tablet per day for 5 days……” (Prescriber 3),

“…I normally explain to my patients how to appropriately use antibiotics… Also, I explain to them the possible side effects when given antibiotics……” (Dispenser 20)

##### Knowledge about AR and its causes

Participants of this study expressed knowledge on the multi-level contributors of antibiotics resistance in pediatrics. They stated that antibiotic resistance is contributed by patients’ behavior on antibiotics’ use, health care providers themselves, and hospital management.

… In the community, there is self-medication… a patient visits a community outlet and requests for a certain antibiotic without any investigation or being attended by a health care provider (Dispenser 2)

Prescribers also contribute to antibiotic resistances through polypharmacy …… Sometimes a patient is given two or three different antibiotics for treating the same infection for the purpose of increasing hospital income…… (Prescriber 3)

… Sometimes in hospitals when antibiotics are about to expire, prescribers are instructed to prescribe them to patients even when there is no clear indication, for example, it has happened mostly with meropenem… (Prescriber 4)

##### Knowledge on the negative impacts of antibiotic resistance

Participants of this study expressed that adequate knowledge on the impacts of antibiotics resistance influence the antibiotics prescription and thus antibiotics use. The latter was expressed as knowledge on cost implication of antibiotics resistance and knowledge on antibiotics resistance as a burden to the patients.

“…Antibiotic resistance is a major concern as it increases cost of treatment and causes complications …. It is a threat to human health and economy; therefore, I think we should take action now.” (Prescriber 2)

“Resistance is problematic to neonates, the first line drugs such as ampicillin plus gentamicin do not respond to neonatal sepsis…the second line drug, like ceftriaxone is very expensive but yet increased resistance to ceftriaxone is reported…” (Prescriber 11)

#### Attitude on antibiotics use in children and AR

##### Positive attitude towards continued medical education

Participants felt that continued training is crucial to improve antibiotics dispensing practices and thus essential in fighting antibiotics resistance. The participants stated that the training helps to improve decision making, influence the behavior of the prescribers/ dispensers, improve the component of dispense-patient communication, knowledge on antibiotic resistance and improves the dispenser's confidence.

“Training helped me to understand the drug interactions……which in turn has helped me to change my prescribing behavior to reduce the spreading of drug resistance…… (Prescriber23)

…Training has helped me in decision making and in educating my colleagues……dispensing is not only about issuing medication but providing instruction to patients on how to use the medication because a slight mistake may harm the patients…….”. (Dispenser 17)

##### Being guided by adequate laboratory investigations

Participants of this study stated that to address antibiotic resistance, it is crucial to have adequate laboratory investigations for coming up with a definite diagnosis before initiating antibiotics. For them laboratory investigations including culture and sensitivity whenever possible were seen to influence rational use of antibiotics and thus reduce antibiotic resistance.

“High quality laboratory investigations should be conducted prior to antibiotic prescription. For example, when a child has a fever or stomach ache and the doctor suspects UTI, an appropriate laboratory should be conducted to guide antibiotic prescription ……”( Prescriber 3)

“Culture and sensitivity tests play a key role in reducing the burden of antibiotic resistance. As it helps in isolating the bacteria responsible for a particular medical condition before the prescription of antibiotics……” (Dispenser 3)

Some participants stated that some of the laboratory investigations are time-consuming and thus they tend to avoid them. For that case they stated that time-consuming investigations challenge the prescribers on which decision to take and often prescribe empirically.

“I normally receive culture and sensitivity test results at least after 72 h …sometimes the patient needs urgent treatment, I therefore initiate antibiotics …… (Prescriber 23)

“……prescribers think ordering culture and sensitivity tests is a waste of time as it takes a long time to get the results…………they see it as a disturbance to ask the patient to return after 48 hours for culture and sensitivity results…” (Dispenser16)

##### Negative attitudes towards private health facilities and community drug outlets

Participants felt that private health facilities were profit-oriented and thus their main focus was not on good outcomes of the patients. They further added that private health facilities were more likely to have poor quality laboratory investigations as their focus was profit-making other than the outcomes of the patients.

“…………. Probably the problem is with Accredited Drug Dispensing Outlets (ADDOs) shops because the patient goes direct and presents his or her symptoms and gets the medications, so it will depend on the knowledge of the dispenser. But remember those are business people so even when a patient complains of flu who may just need paracetamol or cetirizine if the patient requests for amoxicillin they will dispense…………” (Prescriber 3)

“….. Sometimes when parents take their children to private laboratories……most of the private laboratories lack integrity. Even if there are no reagents to diagnose the infection, they will take the sample and release the results” …. (Dispenser 23).

#### Practice on antibiotics use in children and AR

##### Health facility contributors to AR

Participants stated that health facilities contribute to antibiotic resistance through the management pressure that focuses on raising hospital income. They also reported that political pressure and being forced to prescribe based on the available stock contributed to antibiotic resistance.

“Sometimes the hospital management forces us to dispense strong antibiotics without a laboratory test. Sometimes we advise prescribers and they understand but in most cases, we are forced to dispense, if you resist you will be blamed for allowing expensive antibiotics to expire…” (Dispenser 23)

Sometimes…. amoxicillin/clavulanic acid are out of stock, and we receive calls from pharmacies that only certain antibiotics are available… so whoever comes we prescribe what is available, just like that. (Prescriber 2)

##### Health professionals’ practices contributing to AR

Some of the dispensers interviewed in this study stated that some health care providers' practices contribute to the increase of antibiotic resistance. They mentioned some of the practices to include spending less time with patients and thus giving inadequate instruction on the use of antibiotics to the patients.

“Dispensers contribute to AR because they don’t provide enough education to patients before dispensing antibiotics….”. (Dispenser16)

##### Patients practices contributing to AR

Participants viewed patients as a good contributor to the increase in antibiotic resistance. They stated that some patients do not complete the prescribed dose even if they are instructed to complete the dose by a health practitioner. Further-more some patients request for incomplete doses as a result of financial limitations.

“you may prescribe the correct antibiotic but they (patients) will not complete the dose,… after feeling good, they stop taking the drug ……” ( Prescriber 8)

“…. probably because of financial constraints, some patients come with a prescription of a certain antibiotic requesting half a dose ….” (Dispenser 11)

##### Strategies towards reducing antibiotic resistance

Participants of this study highlighted the strategies towards reducing antibiotics resistance that included sustainable continued development education for health professionals, adherence to professional practice in provision of antibiotics, practicing antibiotic stewardship activities and continuous provision of health education to patients on antibiotic use.

“So we try to educate mothers not to self-medicate children at home, rather visit nearby health care facilities…………so we educate them on irrational uses of medicines”. (Prescriber 19)

“I usually educate patients on rational uses of antibiotics… I provide appropriate instructions on how to use the medicines ….” (Dispenser18)

## Discussion

We conducted this study to assess if KAP of prescribers and dispensers could drive irrational use of antibiotics among children in Tanzania. The overall knowledge was found to be adequate in both quantitative and qualitative findings, for example, most of the respondents were aware that misuse of antibiotics can trigger antibiotic resistance. Similar to other reports, knowledge on rational use of antibiotics, the existence of AR and its contributors did not seem to be the issue with majority of healthcare workers [27, 28] rather the practice, findings of this study adds weight on the recommendation of tailored strategies targeting to promote prudent use of antibiotics and availing the much needed information on local AR patterns, to inform the prescribing and dispensing practice [29].

Although few, some prescribers were observed to have poor knowledge on antibiotics. For example, 24% of the prescribers admitted to having a hard time selecting the right antibiotics, 19% were unsure if AR is a worldwide problem and 7.4% didn’t think they ought to know the resistance pattern of the antibiotic before prescribing it. According to Tanzanian treatment guidelines, selection of antibiotics should be informed by culture and sensitivity [30], the reasons clinicians are having a hard time choosing the right treatment could be due to the preference for empirical prescription, this has been observed elsewhere in Tanzania [31]. Drivers for empirical treatment are rooted in the culture of the clinicians themselves [7] and are justified by poor laboratory infrastructure and lack of information on local AR [29] relieving the need to know resistance patterns before prescribing.

From qualitative interviews, participants displayed adequate knowledge on multi-contributors AR and AR impact to the community. Community self-medication of antibiotics was mentioned as one of the main contributors of antibiotic resistance among others. According to Byrne et al., antibiotic use in the community is 4 times that of a hospital set up and most of these antibiotics are being accessed without prescription [32]. In the era of breaching the gap of accessibility of antibiotics in underserved areas, significant challenges are arising due to the readily available antibiotics over-the-counter, the spread of counterfeit medications, and community misuse of antibiotics for animals and agriculture [33]. Community education on the rational use of antibiotics and AR is needed to promote appropriate use of antibiotics. Introducing and enforcing antibiotics regulations should also be considered to reduce antibiotic self-prescription [34].

Moreover, this study found some positive attitudes toward antibiotic use for children. Prescribers and dispensers believed that workshops and training on the rational use of antibiotics is one of the ways of fighting against antibiotic resistance. This is similar to other studies conducted elsewhere [32, 35]. However medical education for this population should be informed by prior identified gaps and tailored to practically suit the current situation in hospital settings.

Apart from the majority, a few prescribers (14.8%) agreed and strongly agreed that prescription of unnecessary antibiotics to a patient does not cause any harm. According to Rezal et al., prescribers tend to prescribe un-required antibiotics in order to avoid more serious superinfection that they might have missed [35]. Furthermore, in order to see more patients in a short period, prescribing antibiotics seems to be an effective way to shorten discussions and conclude an office visit [36]. Moreover, about 33% of dispensers didn’t think individual efforts to implement antimicrobial stewardship would make a difference on the burden of AR. Although antimicrobial stewardship is a collective pursuit [37], individual efforts do count, considering the health care workers to population ratio of 5.2 to 10,000 in Tanzania [38]; One person’s effort can make a difference. Hence education campaigns should encourage individual efforts to foster rational antibiotic use.

Perspectives from qualitative findings on attitude emphasized on the importance of laboratory investigation to guide rational prescription and dispensing of antimicrobials however prescribers had concerns on these investigations taking a long time and hence wasting of their time and bringing disturbance to the patients. According to Polage et al., in Ghana at least 90% of physicians rarely or never ordered laboratory tests because of time constraints [39]. Although most prescribers admitted that they should not prescribe antibiotics without proof of a bacterial infection, in actual practice, they often did not wait for test results before they prescribed [40]. Several paradigms have been reported among clinicians contributing to poor utilization of the laboratory; these include the unreliability of laboratory results [29] and their clinical experience which was more important than any lab investigation [41]. According to Opuku et al. Patients referred for laboratory investigations were 29% less likely to be prescribed antibiotics than those not referred [42]. Evidence-based prescription can halt or slow down the steady increment of AR burden [29], hence there is need to enforce antibiotic stewardship practices. Moreover, motivation to generate profit among private health facilities was also perceived as one of the factors driving irrational use, this is also reported elsewhere in Tanzania [43] and it is evidence of the absence of legal consequences and limited leadership and governance from health policy decision-makers and regulatory bodies [29].

Despite good knowledge and some positive attitudes, we found poor practice, especially among prescribers whereby about 36% of prescribers agreed and strongly agreed to prescribe antibiotics according to the availability of the antibiotic at the facility. Similarly in Uganda prescriptions were based on availability, not suitability [44]. Despite efforts made to improve access to antibiotics, challenges still remain especially in public hospital facilities. Limited government expenditure could in part explain this [45]; also uncoordinated supply chain, procurements not informed by local surveillance of disease prevalence [46], and over-purchase of antibiotics close to expiry [36]. The consequence of poor planning is observed to fall squarely on the prescribers as the management pushes for prescription of only in stock or near-expiry antibiotics [36]. This in turn serves as a major barrier to effective antibiotic use since lab investigation will likely not inform the prescription practice.

Poor Practices among Dispensers were attributed by 29% who sometimes dispensed antibiotics without prescription and 7% and 6% who often and almost always did this. Non-prescription antibiotics are becoming common despite antibiotics being prescription only [33, 45]. An interplay of a number of factors have normalized this, this includes patients’ demand [5], creation of revenue [43] and lack of legal consequence [47]. Another legislative component to prescription-only policy on antibiotics may not be over-emphasized the existing policy [48], rather regular inspections and tailored education campaigns on antibiotic stewardship targeting the community and health care workers.

Findings from a qualitative survey, pointed out patients not receiving adequate instruction from dispensers. Antibiotic dispensing without instructions may lead to wrong self-dosages and inappropriate dosing intervals that might harm the patients [48]. This is also a potential area for educational intervention. The majority of the Tanzanian population is illiterate, hence proper, simplified instructions are essential for rational antibiotic use.

This study was limited to prescribers and dispensers and how they drive irrational use of antibiotics among children, although other health professions such as laboratory scientists and nurses could have some influence on this practice as well. Nonetheless, this does not take away from the fact that findings presented in this study are a representation of the situation in referral regional hospitals in Tanzania. Further studies should aim to study hospital facilities at lower levels and include other health care professions that could have influenced the decision to prescribe or dispense.

## Conclusions

This study found an adequate knowledge, some positive attitude, and poor practice on appropriate antibiotic use and AR for children among prescribers and dispensers. Poor practice was linked to shortage of facilities such as non-functioning laboratories, un-availability of reagents, inadequate number of healthcare providers, limited training on antibiotics use and antibiotics resistance, patients' behaviors (sharing of prescription and incomplete dose), and poverty, among others. This study recommends re-training and instigation of measures to ensure sustainable implementations of antibiotics stewardship programs in regional referral hospitals in Tanzania. Furthermore, regulatory bodies should conduct regular audits on systems influencing antibiotics misuse in RRH to identify loopholes of misconduct and systemic weaknesses that could be fueling the irrational use of antibiotics among children in the country.

## Supplementary Information

**Additional file 1.** Dispensers responses on individual questions.

**Additional file 2.** Prescribers responses on individual questions.

## Data Availability

All datasets on which the conclusions of the paper rely, are provided within this paper.
