# Supplementary material for: Drivers of irrational use of antibiotics among children: a mixed-method study among prescribers and dispensers in Tanzania
Source: BMC Health Serv Res. 2022 Jul 29;22:961. doi: 10.1186/s12913-022-08359-7 (PMC9335991; doi:10.1186/s12913-022-08359-7)
Supplement: Supplementary file 1 — Additional file 1. Dispensers responses on individual questions. [file 12913_2022_8359_MOESM1_ESM.docx]

**Additional file 1. Dispensers responses on individual questions**

**Drivers of irrational use of antibiotics among children:A mixed-method study among Prescribers and Dispensers in Tanzania.**

Lilian Nkinda^1^, Manase Kilonzi^2^, Fatuma F. Felix^2^, Ritah Mutagonda^2^, David T. Myemba^2^, Dorkasi L. Mwakawanga^3^_,_ Upendo Kibwana^1^, Belinda J Njiro^1^, Harrieth P Ndumwa^1^_,_ Rogers Mwakalukwa^2^, Gerald Makuka^1^, Samson W. Kubigwa^5^, Alphonce I. Marealle^2^, Wigilya P. Mikomangwa^2^, Godfrey Sambayi^2^, Peter P. Kunambi^1^, Betty A. Maganda^2^, Nathanael Sirili^4^_,_ Rashid Mfaume^6^, Arapha Bashir Nshau^7^, George M. Bwire^2+^, Robert Scherpbier^8^, Elevanie Nyankesha^9^ Pacifique Ndayishimiye^10*^

**Additional file 1. Dispensers responses on individual questions**

**Supplementary Figure 1. Dispensers Reponses on individual questions on knowledge about antibiotics use in children**

**Supplementary Figure 2. Dispensers responses on individual questions on attitude about antibiotics use in children**

**Supplementary Figure 3. Dispensers responses on individual questions about practice on antibiotics use in children**
