# Supplementary material for: Drivers of irrational use of antibiotics among children: a mixed-method study among prescribers and dispensers in Tanzania
Source: BMC Health Serv Res. 2022 Jul 29;22:961. doi: 10.1186/s12913-022-08359-7 (PMC9335991; doi:10.1186/s12913-022-08359-7)
Supplement: Supplementary file 2 — Additional file 2. Prescribers responses on individual questions. [file 12913_2022_8359_MOESM2_ESM.docx]

**Additional file 2. Prescribers responses on individual questions**

**Supplementary Figure 4. Proportion of prescriber choices on items of antibiotic knowledge scale**

**Supplementary Figure 5. Proportion of prescriber choices on items about attitude**

**Supplementary Figure 6. Proportion of prescriber choices on items about practice**
